# Genome Sequences of Two *Listeria monocytogenes* Strains from Nectarines Associated with Listeriosis in 2014

**DOI:** 10.1128/genomeA.00389-17

**Published:** 2017-07-20

**Authors:** Chunye Lu, Olivera Marjanovic, Christina Morales, David Kiang

**Affiliations:** California Department of Public Health, Food and Drug Laboratory Branch, Richmond, California, USA

## Abstract

*Listeria monocytogenes* is an important foodborne pathogen. Here, we present the annotated whole genome of *Listeria monocytogenes* strains F14M01297-C2 and F14M01297-C4, isolated from nectarines distributed by a packing facility in California during an investigation of listeriosis associated with stone fruit in 2014.

## GENOME ANNOUNCEMENT

*Listeria monocytogenes*, a Gram-positive facultative anaerobe, causes listeriosis, a severe foodborne infection. Listeriosis affects the elderly, newborns, and adults with a weakened immune system. Listeriosis in pregnant women can lead to premature delivery, miscarriage, stillbirth, and health problems for the newborns (1). Listeriosis associated with stone fruits in California was reported by the CDC in 2014 (2) and is the first reported link between human listeriosis and stone fruit. Strains F14M01297-C2 and F14M01297-C4 were isolated during an outbreak investigation from nectarines recovered from a consumer in July 2014 and share the same pulsed-field gel electrophoresis pattern as the clinical isolates. Here, we present the draft genome sequences of isolates F14M01297-C2 and F14M01297-C4.

Genomic DNA from F14M01297-C2 and F14M01297-C4 was extracted from overnight culture using the DNeasy blood and tissue kit (Qiagen, Valencia, CA) following the manufacturer’s instruction. A DNA library was prepared using the Nextera XT kit (Illumina, San Diego, CA). Paired-end sequencing was performed on the MiSeq platform using the MiSeq reagent kit v2 for 500 cycles (Illumina, San Diego, CA), according to the PulseNet USA SOP PNL32. FastQC v0.11.2 (http://www.bioinformatics.babraham.ac.uk/projects/fastqc) was used for basic quality control checks on raw sequencing reads and to calculate coverage. *De novo* assembly of raw reads from MiSeq, trimmed of adaptors, was performed using the SPAdes genome assembler 3.5.0 in Basespace (3, 4). The genome was annotated using the Rapid Annotations Subsystems Technology (RAST) server (5–7) and the Prokaryotic Genome Annotation Pipeline (PGAP) at NCBI (8).

Sequencing yielded a total of 875,350 and 1,082,047 reads for F14M01297-C2 and F14M01297-C4, with an average coverage of 146× and 180×, respectively. The G+C content is 38% for both isolates. Sequencing read assembly yielded a high-quality draft genome consisting of 23 contigs. The total length of the draft genome is 2,914,412 and 2,915,263 bp, with the contig *N*_50_ of 303,357 and 310,079 bp for F14M01297-C2 and F14M01297-C4, respectively. Annotation by the RAST server shows that the draft genome contains 2,854 coding sequences, with 405 subsystems for F14M01297-C2, and 2,855 coding sequences, with 407 subsystems for F14M01297-C4. Both genomes have 22 genes responsible for resistance to antibiotics and toxic compounds, including 2 genes for bile hydrolysis, 3 genes for cobalt-zinc-cadmium resistance, 2 genes for arsenic resistance, 1 gene for fosfomycin resistance, 5 fluoroquinolone resistance genes, 2 beta-lactamase genes, and 2 genes for multidrug resistance efflux pumps. Both isolates contain nine pathogenicity islands including virulence cluster protein A (VclA) and B (VclB), phosphatidylinositol-specific phospholipase C (PI-PLC), broad-substrate range phospholipase C (plcB), actin-assembly inducing protein (ActA) precursor, thiol-activated cytolysin (TACY), and zinc metalloproteinase precursor. The annotations by PGAP are as follows: 2,815 coding sequences, 24 pseudogenes, 56 tRNAs for F14M01297-C2, and 2,814 coding sequences, 27 pseudogenes, 56 tRNAs for F14M01297-C4. Comparative analysis between these isolates and the clinical isolates will be performed in order to reveal the genetic relatedness between these isolates.

### Accession number(s).

This whole-genome shotgun project has been deposited at DDBJ/EMBL/GenBank under the accession no. LFVS00000000 (F14M01297-C2) and LFVT00000000 (F14M01297-C4). The versions described in this paper are versions LFVS02000000 and LFVT02000000, respectively.
